# The SPLENDID Eating Detection Sensor: Development and Feasibility Study

**DOI:** 10.2196/mhealth.9781

**Published:** 2018-09-04

**Authors:** Janet van den Boer, Annemiek van der Lee, Lingchuan Zhou, Vasileios Papapanagiotou, Christos Diou, Anastasios Delopoulos, Monica Mars

**Affiliations:** ^1^ Sensory Science and Eating Behaviour Chair Group Division of Human Nutrition Wageningen University Wageningen Netherlands; ^2^ Electronics & Firmware Systems Division Centre Suisse d'Electronique et de Microtechnique Neuchâtel Switzerland; ^3^ Multimedia Understanding Group Department of Electrical and Computer Engineering Aristotle University Thessaloniki Greece

**Keywords:** chewing sensor, weight management, obesity prevention, overweight, PPG sensor, in-ear microphone, mobile phone

## Abstract

**Background:**

The available methods for monitoring food intake—which for a great part rely on self-report—often provide biased and incomplete data. Currently, no good technological solutions are available. Hence, the SPLENDID eating detection sensor (an ear-worn device with an air microphone and a photoplethysmogram [PPG] sensor) was developed to enable complete and objective measurements of eating events. The technical performance of this device has been described before. To date, literature is lacking a description of how such a device is perceived and experienced by potential users.

**Objective:**

The objective of our study was to explore how potential users perceive and experience the SPLENDID eating detection sensor.

**Methods:**

Potential users evaluated the eating detection sensor at different stages of its development: (1) At the start, 12 health professionals (eg, dieticians, personal trainers) were interviewed and a focus group was held with 5 potential end users to find out their thoughts on the concept of the eating detection sensor. (2) Then, preliminary prototypes of the eating detection sensor were tested in a laboratory setting where 23 young adults reported their experiences. (3) Next, the first wearable version of the eating detection sensor was tested in a semicontrolled study where 22 young, overweight adults used the sensor on 2 separate days (from lunch till dinner) and reported their experiences. (4) The final version of the sensor was tested in a 4-week feasibility study by 20 young, overweight adults who reported their experiences.

**Results:**

Throughout all the development stages, most individuals were enthusiastic about the eating detection sensor. However, it was stressed multiple times that it was critical that the device be discreet and comfortable to wear for a longer period. In the final study, the eating detection sensor received an average grade of 3.7 for wearer comfort on a scale of 1 to 10. Moreover, experienced discomfort was the main reason for wearing the eating detection sensor <2 hours a day. The participants reported having used the eating detection sensor on 19/28 instructed days on average.

**Conclusions:**

The SPLENDID eating detection sensor, which uses an air microphone and a PPG sensor, is a promising new device that can facilitate the collection of reliable food intake data, as shown by its technical potential. Potential users are enthusiastic, but to be successful wearer comfort and discreetness of the device need to be improved.

## Introduction

### Background

The available methods for monitoring food intake—which for a great part rely on self-report—often provide biased and incomplete data [1-5]. Depending on the exact method used, they require people to eat consciously, be knowledgeable about what they eat, be able to estimate portion size, and remember all that information. As a result, these methods are prone to underreporting. It is common for people to report an unrealistically low energy intake, that is, an energy intake that is too low to sustain their body at a low level of physical activity [6-9]. Current technological advances have enabled the development of tools that can facilitate the collection of reliable food intake data.

Currently, some devices are available that can be used to increase the reliability of food intake monitoring. The Mandometer, for example, can be used to measure the size of meals. It is a weighing scale that is placed underneath the plate during a meal [10]. Furthermore, a number of wearable devices have been developed that can automatically detect eating [11-14]. These are mostly ear- and neck-worn devices. They use sensors (eg, a microphone or strain sensor) to collect signals that contain information on whether or not a person is eating. Pattern-recognition algorithms are used to extract this information.

In particular, devices that can detect eating events have the potential to reduce underreporting. Such a device can take away the need for people to be conscious about their eating. Moreover, this information can be used to prompt people to report what they are eating at the moment they are eating it. It can, therefore, also take away the need for people to remember what they ate. However, there is not yet a device for the automatic detection of eating that is practical for everyday use, despite the progress made in this area. Such devices, for example, require people to accurately position a sensor on the body with tape or require people to wear items like glasses or a hat to carry the functional parts [13,15,16].

### Development of the SPLENDID Eating Detection Sensor

Within the context of SPLENDID, an information and communications technology project funded by the European Union [17,18], we aimed to take the next step and develop a device for the automatic detection of eating events that is practical for everyday use. It was decided to create an ear-worn device as this was expected to be acceptable for young, overweight adults, which was our primary target group. In the future, such a device could be incorporated into other devices the target group is already using, such as earphones used for listening to music. Moreover, such a device could be appropriate for a wider population.

The eating detection sensor was built using an iterative, incremental development approach. At each iteration (ie, development stage), we introduced design modifications, added new functionalities, and evaluated the resulting prototype. The development of the eating detection sensor consisted of 3 stages. These are briefly described below.

#### Development of Preliminary Prototypes

During the development of the eating detection sensor, different options for signal collection were considered:

- An air microphone placed at the beginning of the ear canal that measures sounds produced by chewing [19-21].
- A bone conduction microphone placed on the cheekbone just in front of the ear that measures the vibrations in the bone produced by chewing [22,23].
- A photoplethysmogram (PPG) sensor placed on the ear that measures the blood volume in the tissue of the ear, which is affected by chewing activity [20,21,24]. This technique has never before been used for this application.

For all three options, a prototype was developed, and these prototypes were tested in a laboratory study [19].

#### Development of the First Wearable Version

Based on the results of the laboratory study, we decided to continue with a combination of the air microphone and PPG sensor without the bone conduction microphone. Overall, the air microphone had shown the best results, but the PPG sensor was better at detecting soft foods [19]. These two sensors were combined for more accurate detection of eating events over a wide range of foods. Furthermore, due to its low sampling rate (21.33 Hz for our prototype), the PPG sensor has low battery requirements and is computationally efficient.

To make the new version of the eating detection sensor wearable, another device the “datalogger” was added to it [21]. It houses a data acquisition system, a battery, and an accelerometer. It is connected via a cable to the eating detection sensor and is worn in the trouser pocket or on a belt.

This first wearable version of the eating detection sensor was tested in a semicontrolled study [20,21,24]. The results obtained with the eating detection sensor were promising and were further improved by the addition of an accelerometer in the datalogger. Algorithms using signals from the air microphone, PPG sensor, and accelerometer achieved an accuracy of 0.938, a precision of 0.794, and a recall of 0.807 [21].

#### Development of the Integrated Version

**Figure 1:**
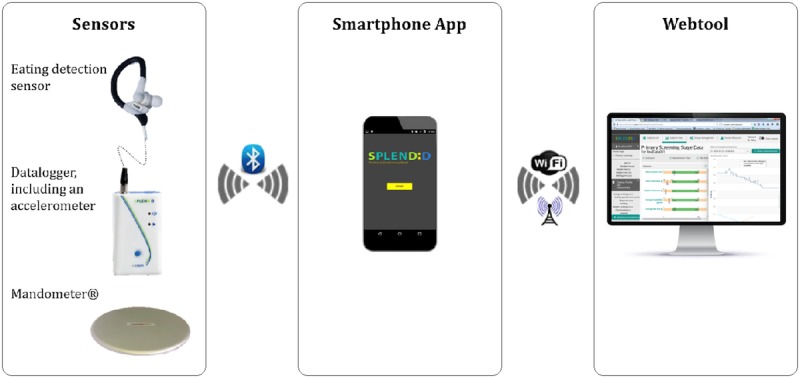
The SPLENDID eating detection sensor integrated into the full SPLENDID system. This system combines the eating detection sensor with a datalogger (including an accelerometer), the Mandometer, a smartphone app, and a webtool, functioning together as a “wearable personal coach.”.

Finally, the wearable eating detection sensor was integrated into a larger system for added functionality (Figure 1). This system includes, among others, a smartphone app and a webtool. The smartphone app can prompt the user to report the detected eating events. The webtool can provide an overview of the recorded eating events. Furthermore, goals regarding a healthy eating pattern can be entered into this webtool. Consequently, the smartphone app can help the end user achieve these goals by providing real-time feedback when the eating detection sensor is worn. The integrated version of the eating detection sensor was tested in a 4-week feasibility study.

### This Study

In this study, we aimed to explore how potential users perceive and experience the SPLENDID eating detection sensor. This will offer insight into its feasibility from a user’s perspective. Furthermore, this will provide directions for the further development of the SPLENDID eating detection sensor and the development of similar devices. During the development of such devices, the primary focus is usually on their technical performance, but for these devices to be successful, they also need to be acceptable to the users.

**Figure 2:**
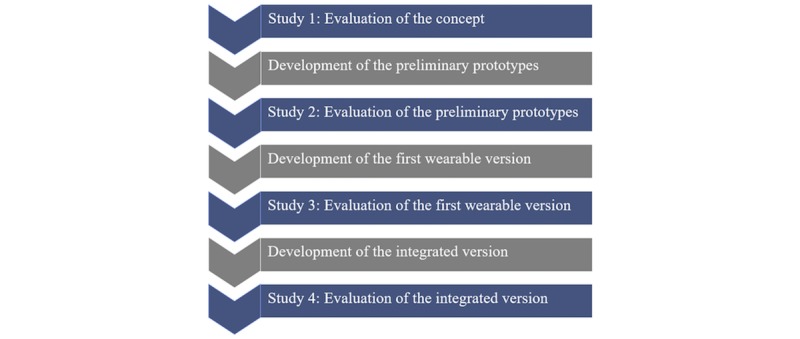
Flowchart indicating how the evaluation studies (blue) relate to the development stages of the SPLENDID eating detection sensor (gray).

Potential users evaluated the SPLENDID eating detection sensor at the different stages of its development (Figure 2).

- Study 1, evaluation of the concept of SPLENDID eating detection sensor: Before any prototypes of the eating detection sensor were developed, health professionals (n=12) were interviewed, and a focus group was held with potential end users (n=5) to find out their thoughts on the concept.
- Study 2, evaluation of the preliminary prototypes of the eating detection sensor: Young, normal-weight adults reported their experiences with the three preliminary prototypes of the eating detection sensor during the laboratory study.
- Study 3, evaluation of the first wearable version of the eating detection sensor: Young, overweight adults reported their experiences with the subsequent version of the eating detection sensor during the semicontrolled study where they used the sensor on 2 separate days (from lunch till dinner).
- Study 4, evaluation of the integrated version of the eating detection sensor: Finally, young, overweight adults reported their experiences with the eating detection sensor during a 4-week feasibility study where they used the eating detection sensor in combination with other devices (see Figure 1).

## Methods

### Study 1: Evaluation of the SPLENDID Eating Detection Sensor Concept

Before any prototypes of the eating detection sensor were developed, potential users were asked about their thoughts on the concept of the eating detection sensor. Health professionals were interviewed, and a focus group was held with potential end users.

#### Study 1a: Interviews with Health Professionals

We conducted semistructured, in-depth, face-to face interviews with 12 health professionals who deal with weight management professionally (eg, dieticians, personal trainers). First, the concept was explained to them, and subsequently, they were asked about their views on different aspects of the concept. All interviews were recorded and later transcribed and systematically analyzed.

#### Study 1b: Focus Group With Potential End Users

A focus group was held with 5 young women (mean age 22 [SD 2] years; mean body mass index [BMI] 22.5 [SD 1.9] kg/m^2^) interested in weight management. First, the concept was explained to them, and subsequently, they were asked open-ended questions to facilitate discussion. The focus group was recorded and later transcribed and systematically analyzed.

### Study 2: Evaluation of the Preliminary Prototypes of the Eating Detection Sensor

**Figure 3:**
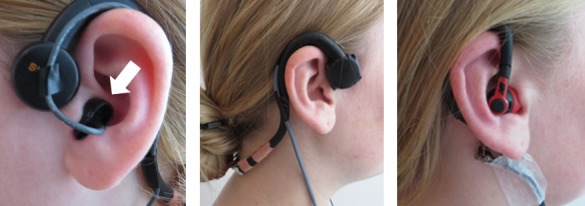
Preliminary prototypes of the eating detection sensor: air microphone (left), bone conduction microphone (middle), photoplethysmogram sensor (right).

The preliminary prototypes of the eating detection sensor were tested in a laboratory setting [19]. The pictures of these prototypes are shown in Figure 3. With these prototypes, it was yet not possible to move around freely.

The prototypes were tested by 23 healthy, young adults (13 men and 10 women; age 23 [SD 3] years; mean BMI 22.6 [SD 3] kg/m^2^). They visited the university for a test session of approximately 1.5 hours. During this session, all three prototypes were worn simultaneously by the participants while they consumed a variety of foods and performed other activities such as talking. The air microphone was worn on the left ear, and the bone conduction microphone and PPG sensor were worn on the right ear. Afterward, the participants received a questionnaire concerning their experiences with the sensors. This included both closed and open-ended questions.

This study was approved by the Medical Ethical Committee of the Wageningen University (NL 48839.081.14).

### Study 3: Evaluation of the First Version of the Wearable Eating Detection Sensor

**Figure 4:**
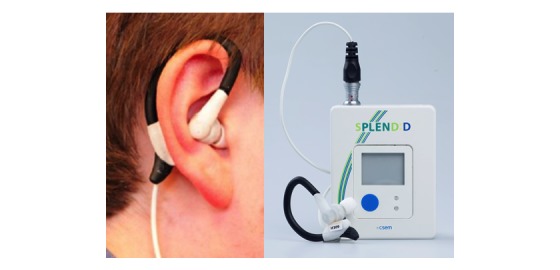
First wearable version of the eating detection sensor (left: the eating detection sensor; right: the eating detection sensor and the datalogger).

One year later, the first wearable version of the eating detection sensor was tested in a semicontrolled study [20,21,24]. It comprised a commercial earhook in which both the air microphone and PPG sensor were incorporated (Figure 4). Also, a magnet was included to ensure that the PPG sensor was positioned properly. Furthermore, as described in the introduction, the datalogger was added to the eating detection sensor to make it wearable (Figure 4). The combination of the air microphone, PPG sensor, and accelerometer (incorporated into the datalogger) enables more accurate detection of eating events [21].

Twenty-two overweight, young adults (3 men and 19 women; mean age 23 [SD 2] years; mean BMI 28.0 [SD 2.3] kg/m^2^) tested the wearable eating detection sensor. They participated for 2 testing days. They arrived just before lunch (11 am) and left after they had dinner (around 6 pm). At these testing days, they performed common, daily-life activities (including snacking) while wearing the eating detection sensor. Furthermore, the participants completed questionnaires on user comfort, which included both closed and open-ended questions.

This study was approved by the Medical Ethical Committee of the Wageningen University (NL52100.081.15).

### Study 4: Evaluation of the Integrated Version of the Eating Detection Sensor

**Figure 5:**
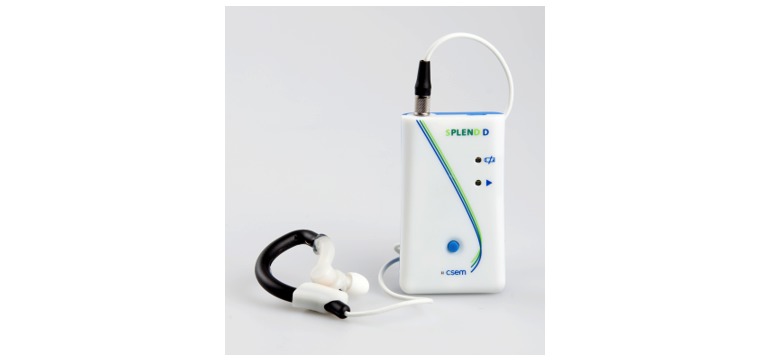
Integrated version of the SPLENDID eating detection sensor with its datalogger.

**Figure 6:**
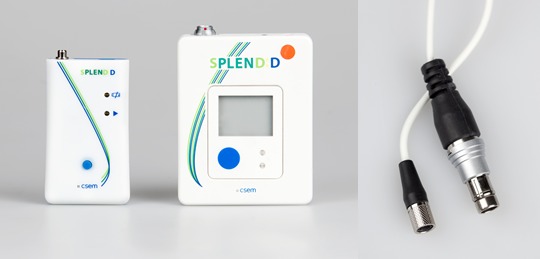
Old version and new, smaller version of the datalogger (left) and plug (right).

Finally, the integrated version of the eating detection sensor was tested by young, overweight adults in a 4-week feasibility study (Figures 1 and 5). To increase wearer comfort, the size of the datalogger and plug was reduced in this version (Figure 6). The eating detection sensor was virtually unchanged, and because of known issues with wearer comfort, the participants only had to wear it for 2 hours per day.

In total, 20 overweight, young adults (4 men and 16 women; mean age 25 [SD 2] years; mean BMI 28.8 [SD 2.8] kg/m^2^) motivated to adopt healthier behavior participated in the 4-week feasibility study. During the first week, the participants used the system to assess their baseline eating behavior. Based on the observed behavior, personal goals were set for the following 3 weeks regarding the number of snacks. During these 3 weeks, the participants received personalized feedback through the smartphone app to help them achieve these goals. Afterward, they completed a questionnaire on their experiences, which included both closed and open-ended questions.

This study was approved by the Medical Ethical Committee of the Wageningen University (NL56853.081.16).

## Results

### Study 1: Evaluation of the SPLENDID Eating Detection Sensor Concept

#### Study 1a: Interviews With Health Professionals

A device like the eating detection sensor was new to all health professionals (n=12), but some already had experience with an app (n=5) or accelerometer (n=4). In general, the health professionals were enthusiastic about the eating detection sensor. Some were a bit skeptical at first (n=4), but after talking and thinking about it a little more, they thought that the sensor could be very useful to gain insight into the users’ eating pattern. The users, however, need to forget that they are wearing the eating detection sensor.

The first thing I thought was: this is a bit excessive…But when thinking about myself when I am for example cooking, I unconsciously eat some food. People forget to write that down, so this could be very useful.

An important thing is that end users should “forget” that they are wearing it. Then you will get a good overview of their eating patterns.

Furthermore, they stressed that the eating detection sensor should be reliable and accurate, not cost them too much time, and come with a clear protocol on how to work with it.

#### Study 1b: Focus Group With Potential End Users

The participants were already familiar with all kinds of smartphone apps to record food intake. They were enthusiastic about what the eating detection sensor had to add. One of the participants mentioned that it will help her when she “secretly” eats something, and this will give a good insight into her eating pattern. However, the participants also had some concerns regarding the eating detection sensor. It should be ensured that it is comfortable to wear for a long time, and it should not be too noticeable.

### Study 2: Evaluation of the Preliminary Prototypes of the Eating Detection Sensor

For wearer comfort, the air microphone received an average grade of 6.7 (range: 2-9) on a scale of 1 to 10, the bone conduction microphone 5.8 (range: 2-10), and the PPG sensor 6.7 (range: 4-9). The grade for wearer comfort did not differ significantly between the prototypes (analysis of variance [ANOVA], *P*=.13). The participants indicated they would be able to wear the air microphone for an average of 5.7 (range: 0-24) hours per day, the bone conduction microphone for 5.6 (range: 0.5-24) hours per day, and the PPG sensor for 5.4 (range: 4-9) hours per day. This value did not differ significantly between the prototypes (ANOVA, *P*=.99).

The open-ended questions provide an explanation for these results. The most frequently mentioned remarks regarding the air microphone were that the sensor was comfortable to wear (n=18), but that it lowered the users’ hearing ability (n=10) and that they would get tired of the sensor after wearing it for a longer period (n=15). Regarding the bone conduction microphone, the participants most frequently mentioned that it remained unnoticed while wearing (n=10), that the sensor could be annoying during exercise (n=9), and that the sensor put pressure on their head and neck (n=5). Regarding the PPG sensor, the participants most frequently mentioned that they did not notice that they were wearing the sensor (n=11), the sensor lowered their hearing ability (n=6), and the sensor cable was pulling and annoying (n=4). Regarding the prototypes in general, the most frequently mentioned barrier for wearing them in real life was that they were very noticeable and oddly shaped (n=8). In turn, the most often mentioned wishes were that the prototypes should be as invisible as possible (n=13) and that they should be comfortable to wear (n=10).

### Study 3: Evaluation of the First Wearable Version of the Eating Detection Sensor

The participants graded the wearer comfort of the chewing sensor an average of 3.8 (range: 2-7) on a scale of 1 to 10. Furthermore, participants indicated that they would be able or willing to wear the chewing sensor for 3.9 (range: 2-7 h) hours per day. Some participants, however, mentioned that they would be able to wear it for a longer time if there were breaks in between.

There was large variation in the answers of the participants regarding how the chewing sensor affected eating, moving, and talking. Most participants agreed with the statement that the chewing sensor was bothering them: 19 out of the 20 participants scored higher than 5 on a 9-point Likert scale (1=totally disagree, 5=neutral, 9=totally agree).

The open-ended questions provide an explanation for these results. The most frequently mentioned remarks regarding the wearer comfort of the eating detection sensor were: “the chewing sensor was painful to the ear” (n=16), “the cable was annoying or hindering” (n=14), “the sensor reduced hearing” (n=8), and “internal noises were heard better” (n=5). Three participants experienced no or only little discomfort.

### Study 4: Evaluation of the Integrated Version of the Eating Detection Sensor

Of the 20 participants, 19 experienced discomfort from the eating detection sensor; they started experiencing discomfort after an average of 1 hour and 20 minutes. The participants graded the average wearer comfort of the eating detection sensor at 3.7 (range: 1-7) on a scale of 1 to 10. Moreover, they scored the statement “The sensor bothered me” an average of 5.5 (range: 4-7) on a scale of 1 (totally disagree) to 7 (totally agree).

The participants reported having used the eating detection sensor on an average of 19 out of the intended 28 days, of which they used it for at least 2 hours on 17 days. During the first week, compliance was highest, with the eating detection sensor being used for an average of 6 days. The most frequently mentioned reasons for wearing the sensor <2 hours per day (open-ended question) were discomfort (n=14) and technical issues, such as broken sensor (n=8; Table 1). Furthermore, if the participants used the sensor, it was for an average of 1.9 hours (range: 1-4 hours).

Regarding reactions from the social environment, the participants gave mixed results. They scored the statements “People in my environment noticed the sensor” and “I did not like it when people noticed the sensor” an average of a 3.4 (range: 1-7) on a scale of 1 (totally disagree) to 7 (totally agree).

**Table 1:** Reasons mentioned for wearing the eating detection sensor <2 hours and their frequency.

Reason	Frequency
Discomfort	14
Technical issues (eg, broken sensor)	8
Reduced hearing	6
Impractical (eg, with sports)	6
Inappropriate (eg, at work)	3
Noticeable	1
Forgotten	1
Not enough time	1

**Table 2:** Additional remarks regarding the eating detection sensor and their frequency.

Additional remarks	Frequency
Cable is not practical	7
The eating detection sensor got noticed	7
The eating detection sensor reduced hearing	5
The eating detection sensor was uncomfortable	4
Experienced technical issues with the eating detection sensor	4
Had to explain what the eating detection sensor is	3
Inappropriate to use in certain situations	3
Added value of eating detection sensor unclear	3
Received no reactions from environment	2
Received positive reactions from environment	2
Experienced no problems	2
Looks like listening to music	2
Not practical	1

When the participants were asked whether they had any additional remarks (ie, open-ended question), they most frequently mentioned that the cable was not practical (n=7), the sensor got noticed (n=7), and the sensor lowered their hearing (n=5; Table 2). Furthermore, some participants indicated that they did not see the added value of the sensor because they believed that they did not need it to remind them to note the foods consumed and that the detections were not always accurate.

## Discussion

### Principal Results

The current paper explores how potential users perceive and experience the SPLENDID eating detection sensor. Across the different stages of development, the potential users were enthusiastic about the concept. They especially liked that it provided objective information on their eating patterns. However, they stressed that it needed to be comfortable to wear and discreet. The latest version of the eating detection sensor did not yet meet these requirements.

For the eating detection sensor to meet the user requirements, further improvements need to be made. In particular, the wearer comfort of the sensor requires attention. After wearing the sensor for a while (ie, on average, after 80 minutes) the potential users started experiencing discomfort. As a result, they graded the wearer comfort of the sensor at 3.7 on a scale of 1 to 10 in the final study. Moreover, the experienced discomfort was the main reason for the participants to wear the sensor for <2 hours.

One option would be to offer different shapes and sizes of the eating detection sensor so that the users can find a sensor with a good fit. This would also improve the ability of the device to detect eating events. The current eating detection sensor fits some people better than others, which is reflected in the wide range in the grades for wearer comfort (1-7 for the last version, on a scale from 1 to 10). Another option would be to reduce the size of the eating detection sensor and to make it more like a hearing aid; these are made to be worn throughout the day, unlike earphones. However, the technically feasibility needs to be investigated.

By resolving the issues related to wearer comfort, the visibility of the eating detection sensor is likely to be reduced as well. Furthermore, the visibility of the current version of the eating detection sensor was already acceptable to some of the participants. They mentioned that even though people in the environment noticed the eating detection sensor, they did not recognize it as such because it looks like a device for listening to music. This is a major advantage of the ear-worn devices over some of the other devices that are being developed for the detection of eating events (eg, neck-worn devices or a device mounted onto eyeglasses)[14,25-27].

It would be interesting to repeat the feasibility study once the eating detection sensor has been improved for wearer comfort and visibility. The SPLENDID eating detection sensor is a device with great potential as shown by its technical performance [19-21,24]. It could help provide a more complete picture of food intake, which is a major issue with the current methods for monitoring food intake [1-4,6-9].

### Limitations

In the feasibility study, due to the issues with wearer comfort, the participants were asked to wear the eating detection sensor for at least 2 hours, while it is intended to be used throughout the day. This will affect the user experience. As was mentioned by the health professionals, people need to forget that they are wearing the eating detection sensor. Because the participants only used the eating detection sensor for an average of 1.9 hours per day and started to experience some discomfort after a while, they might not have been able to forget that they were wearing the eating detection sensor.

If the participants in the feasibility study had been less conscious about the fact that they were wearing the eating detection sensor, they probably would also have been less conscious about their eating, and then, the added value of the eating detection sensor would have been more evident. For 15% (3/20) of the participants, the added value of the eating detection sensor was unclear. They did not feel that they needed such a sensor to remind them to report the foods consumed.

### Comparison With Prior Works

To our knowledge, this is the first paper to describe how an ear-worn device for the detection of eating events is received by potential users and to describe their experiences with such a device in real life. It shows that ear-worn devices for the detection of eating events need to meet high standards to be acceptable for everyday use.

When tested in a laboratory setting, the eating detection sensor received a sufficient grade for wearer comfort, while it received an insufficient grade when it was tested in real life. Moreover, the participants did not experience discomfort as soon as they started wearing the sensor; they started experiencing discomfort only after 80 minutes of wearing it. It is important to keep this in mind when interpreting the results from the laboratory studies.

### Conclusions

The SPLENDID eating detection sensor is a promising new device that can facilitate the collection of reliable food intake data as shown by its technical potential, which has been described before. Furthermore, potential users are enthusiastic about it. They especially like that it provides objective information on their eating patterns. However, to be successful, the wearer comfort and discreetness of the device need to be improved. Therefore, further development of the device should mainly focus on the design of the hardware.

## Abbreviations

PPG: photoplethysmogram
